# Multimorbidity, low income and unmet need for healthcare: a national study of 41,135 older adults

**DOI:** 10.1093/eurpub/ckac129.503

**Published:** 2022-10-25

**Authors:** F Alemu, J Thornton, S Ali

**Affiliations:** 1 Department of Epidemiology and Biostatistics, Western University, London, Canada; 2 Department of Family Medicine, Western University, London, Canada

## Abstract

The aims of this study were: (1) to identify the determinants of unmet need for access to primary care in middle-aged and older adults; and (2) to examine the reasons for unmet need. We used data from the Canadian Longitudinal Study on Aging (CLSA), a nationally representative survey of adults aged 45 to 85 years. Respondents were asked if they ‘needed health care during the last 12 months but did not receive it’. For those who replied ‘Yes’, the survey asked for the reason(s) for not receiving the needed care. Out of 41,135 respondents, 3,468 had unmet need for healthcare in the last 12 months. Among respondents with 0, 1, 2 and ≥3 morbidities, the proportion reporting unmet need was 2.5%, 5.3%, 5.1% and 9.1% respectively. After adjusting for covariates, the odds ratios (ORs) for unmet need for 1, 2 and ≥3 morbidities (compared to no morbidity) were 1.25 (95% CI: 0.87 to 1.78; p = 0.23), 1.57 (95% CI: 1.13 to 2.17; p < 0.05) and 2.03 (95% CI: 1.51 to 2.73; p < 0.05), respectively. For income groups, the ORs for unmet need (compared to >$150,000/year) were 0.94 (95% CI: 0.79 to 1.12) for $100,000-$150,000, 1.02 (95% CI: 0.87 to 1.20;) for $50,000-$100,000, 1.30 (95% CI: 1.09 to 1.55) for $20,000-$50,000, and 1.39 (95% CI: 1.08 to 1.78) for < $20,000. Other statistically significant determinants of unmet need included age (older adults were less likely to have unmet need), sex (females were more likely), having a family physician (less likely) and perceived physical and mental health (poor health more likely to be associated with unmet need). The most common reasons for unmet need were: ‘long wait time’ (52.1%) and ‘doctor did not think it was necessary’ (16.7%). Multimorbidity and low-income are associated with higher odds of unmet need among older adults. This disparity is partly due to not having a regular family physician and long wait time to see a doctor. Reducing these barriers are critical to reducing inequalities in health outcomes.

